# Role of Bisphosphonate Therapy in Patients with Osteopenia: A Systemic Review

**DOI:** 10.7759/cureus.4146

**Published:** 2019-02-27

**Authors:** Shumaila M Iqbal, Iqra Qamar, Cassandra Zhi, Anum Nida, Hafiz M Aslam

**Affiliations:** 1 Internal Medicine, University at Buffalo / Sisters of Charity Hospital, Buffalo, USA; 2 Internal Medicine, Brigham and Women's Hospital, Boston, USA; 3 Internal Medicine, Drexel University College of Medicine, Philadelphia, USA; 4 Internal Medicine, Hackensack Meridian School of Medicine at Seton Hall University, Trenton, USA

**Keywords:** osteopenia, bisphosphonate therapy, fractures, bone mineral density

## Abstract

By contrast to clinical trials exploring osteoporosis, clinical trials specifically designed for the osteopenic population are limited. Thus, less clinical data are available regarding treatment benefits and cost-effectiveness of treating a patient population with a bone mass density in the osteopenic range (T-score between -1 and -2.5). In this article, we aimed to highlight this high-risk population with a low bone mass density (BMD) susceptible to high fracture risk by reviewing different national and international guidelines for treating osteopenia. The cost-effectiveness of the therapy for the above-mentioned patient population is also discussed. By reviewing different clinical trials, we have specifically highlighted the role of bisphosphonate therapy for fracture risk reduction and increment in bone mineral density (BMD) in patients with osteopenia.

## Introduction and background

Bone mass density (BMD) is an indicator of bone health and strength. It is a good measure for determining risk of future fractures and an estimation of the body’s response to osteoporotic/osteopenic treatment. In clinical practice, dual-energy X-ray absorptiometry (DXA) is a widely used technique to detect BMD by tracking serial changes. In regards to the DXA measurements, the World Health Organization (WHO) defines osteopenia as having a BMD T-score between -1 and -2.5 at the hip or spine [1-2]. BMD in osteopenia is low enough to be categorized as abnormal but not low enough that would put bony tissue into the osteoporotic range (T score <-2.5). The rate of developing fragility fractures should be higher with lower BMD, indicating a negative correlation. However, most patients presenting with fracture have BMD in the osteopenic range rather than the osteoporotic range [2-3].

Various treatment modalities have been recommended for low bone mass (T-score -1 to -2.5) including nutritional support with adequate intake of Vitamin D and calcium supplementation, lifestyle modifications (smoking cessation and exercise), and prevention of household falls by less tranquilizer consumption, visual aids, or removal of carpets from the floor. Literature is evident for data regarding prevention of osteoporotic fracture with pharmacological therapy; however, treatment for prevention of fractures in patients with osteopenia is limited and most of the clinical trials performed were specifically designed for use in the osteoporotic population. Pharmacological intervention for osteopenia also remains controversial when it comes to weighing the benefits against the cost-effectiveness of the therapy. The review article discussed below will highlight the role of bisphosphonates in reducing the risk of fractures for the patients with a BMD T-score >-2.5.

## Review

Identification of high-risk population susceptible to low BMD-associated fracture

As mentioned previously, BMD is an accurate measure of bone strength and predicts fracture risk. However, fragility fractures are found to be more prevalent with osteopenia rather than osteoporosis. This is true because the number of subjects at risk is more in the zone of having BMD T-scores in the range of -1 to -2.5 rather having BMD <-2.5 [2,4]. These were results quoted in a large clinical trial performed by Siris et al., which obtained data from 34 different states. Results showed four times the risk of fracture in osteoporosis as compared to healthy patients (rate ratio, 4.03; 95% confidence interval [CI], 3.59-4.53). Osteopenia was associated with almost a two-fold higher rate (95% CI, 1.49-2.18) as compared to healthy patients [5]. It shows BMD is not the only factor that determines future risk of fracture and there might be other clinical risk factors that make a subject more vulnerable to high risk of fracture. Identification of those risk factors is thus highly crucial in order to be more precise with risk assessment.

Fracture risk assessment tool

In order to be more precise with estimating fracture risk, WHO in the year 2008 had developed the fracture risk assessment tool (FRAX) that takes into account the important risk factors, thereby making subjects more vulnerable towards fracture. End results analyzed by FRAX were a 10-year risk percentage of a major osteoporotic fracture (MOF) and a 10-year risk percentage of a hip fracture [6]. The risk factors acknowledged by the FRAX tool include age, sex, height, history of previous fractures, maternal hip fracture, glucocorticoids, excessive alcohol consumption, rheumatoid arthritis and secondary osteoporosis [6]. Through the implementation of FRAX, a high-risk population for fracture can be identified in a clinical setting. It is the most widely used calculator by clinicians worldwide and its predictability has been evaluated in the patient population of many different nations. A large population-based cohort study of 141, 320 women was conducted in Israel. Retrospective FRAX scores were calculated using computerized health records and compared with the actual incidence of major osteoporotic fractures during the following 10 years. Results showed BMD exhibited lower predictive performance for both MOF (area under curve = 0.62 vs. 0.65; *P* = 0.003) and hip fractures (area under curve = 0.78 vs. 0.84; *P* < 0.001) as compared with FRAX only [7]. Another study performed in Europe showed that FRAX with BMD and to a lesser extent also without BMD predict major osteoporotic and vertebral fractures in the general population [8]. This is contrary to the study conducted in Japan which found that FRAX predictability for fracture risk was found not to be significantly different from that predicted by BMD only [9]. A separate study conducted in Spain showed good predictability of FRAX without BMD for hip fracture though for major osteoporotic fracture predictability was low [10]. It is important to note that FRAX underestimates future fracture risk as it reports risk for only hip and major fractures, which comprise approximately half of all fragility fractures. FRAX also underestimates risk in patients with multiple osteoporosis-related fractures, recent fractures, lumbar spine BMD much lower than femoral neck BMD, secondary osteoporosis, and increased risk of falling.

Whom to treat pharmacologically?

Once the assessment of high-risk patient population for fragility fractures is made, it is important to consider which subjects would benefit the most from pharmacological treatment for osteopenia. Different guidelines are available to this regard. The year 2014 guidelines of the National Osteoporosis Foundation emphasized pharmacological treatment in postmenopausal women and men age 50 years and older with low bone mass (T-score between -1.0 and -2.5, osteopenia) having 10-year hip fracture probability ≥3 % or a 10-year MOF probability ≥20 % based on the United States of America-adapted WHO absolute fracture risk model [11]. This also coincides with recommendations by the American Association of Clinical Endocrinologists and the American College of Endocrinology Clinical Practice [12]. Additionally, they also recommended pharmacological treatment for osteopenia or low mass density if prior history of fragility fracture of hip or spine is present [12]. The American College of Physicians (ACP) guideline from the year 2017 leans more towards physician clinical judgment in deciding whether to treat or not the osteopenic women of age 65 years and older who are at a high risk of fracture based on their individual fracture profile and personal preference [13]. This recommendation by the ACP was graded as weak due to the low quality of evidence available [13-14].

Pharmacological management of osteopenia

The last two decades have remained an era for the emergence of new pharmacological interventions that would be effective and safe for utilization in subjects with low BMD. The intervention has been broadly classified into those that prevent bone reabsorption (antiresorptive or anti-activation drugs) and those that increase bone formation (anabolic regimens). Studied anabolic regimens includes management with parathyroid hormone (PTH; both intact and truncated), growth hormone, insulin-like growth factor, elemental supplements (strontium, calcium and fluoride), growth factors (vasculoendothelial growth factors, fibroblast growth factors, transforming growth factor beta), statins, and bone morphogenetic protein-2 and 7 [15-16]. Though many have been studied, the only currently available regiments for clinical use, approved by the Food and Drug Administration (FDA) is truncated PTH (1,34 PTH). This includes Teriparatide (brand name: Forteo, approved in the year 2002) and Ablapoparatide (brand name: TYMLOS, approved in the year 2017) [17-19]. PTH increases BMD and decreases the risk of vertebral and non-vertebral fractures (70% and 45%, respectively). However, no evidence was available regarding the prevention of hip fractures by PTH [20]. They are considered the second line of therapy and should be considered if there are contraindications, intolerability, and failure to anti-resorptive drugs. They are also found to be beneficial for severe osteoporosis and osteoporosis induced by glucocorticoids [18]. A third anabolic treatment entity current being under review by the FDA is the monoclonal antibody romosozumab [18]. It acts against sclerostin protein whose function is to inhibit bone formation.

Anti-resorptives are classified into five main groups: bisphosphonates, estrogens, selective estrogen receptor modulators (SERMs), calcitonin and monoclonal antibodies such as denosumab (DNS) [21]. Among these, bisphosphonates and DNS are considered as first-line treatments. Studies have shown increases in BMD with bisphosphonates and DNS. However, BMD of the lumbar spine, total hip, femoral neck, and one-third radius were significantly increased in the DNS group as compared to the bisphosphonates group [22-23]. Though DNS is superior to bisphosphonates for increasing BMD, neither was found to be superior over the other for fracture risk reduction. This has been proved by different meta-analysis and randomized control trials [23-25]. All bisphosphonates are equally effective in fracture risk reduction. However, when compared to placebo, zoledronate has the highest superiority among all bisphosphonates in providing the highest risk reduction for vertebral fractures [25].

The literature recommends administering anabolic synthesized PTH for a single course lasting for 24 months followed by an anti-resorptive to maintain BMD further [20]. A meta-analysis performed by Shenghan et al. showed a significant positive change in BMD of total hip and femoral neck with concomitant anti-resorptive therapy and anabolic therapy (intact and truncated PTH) utilization for an optimal period of therapy (12 months). However, the study did not show a significant advantage in terms of improvement in the BMD of the spine with concomitant therapy [26]. Another article by Cosman F et al. supports the combination therapy as well [27].

Risk reduction of fractures with bisphosphonates

Though risk identification for fracture in osteopenic patients is convenient with the FRAX tool, the availability of data is very limited in terms of determining the effectiveness of the pharmacological intervention. Thus to provide extensive discussion here for highlighting the role of bisphosphonates for fracture risk reduction, post hoc analysis approach was used. Since most of the trials were designed mainly for the osteoporotic population, we have utilized the part of the data that analyzes women with low BMD (the non-osteoporotic population).

A recently conducted large, double-blinded clinical trial involving 2000 women was specifically designed to evaluate the incidence of fractures in osteopenic women of age more than 65 years treated with 5 mg zoledronate vs. placebo. Overall, fracture incidence was significantly lower in the zoledronate group as compared to the placebo group (hazard ratio with zoledronate, 0.63; 95% confidence interval, 0.50 to 0.79; *P *< 0.001). Significant results were achieved for both vertebral (odds ratio, 0.45; *P* = 0.002) and non-vertebral (hazard ratio, 0.66; *P* = 0.001) fragility fractures [28]. The Fosamax International Trial (FOSIT) involved post-menopausal women with BMD -2 or less belonging to 34 different countries. Endpoints were safety, tolerability, and effects of alendronate on BMD and incidence of non-vertebral fractures. The incidence of non-vertebral fractures was significantly lower in the medication group vs. the placebo group. Overall, there was a 47% risk reduction for non-vertebral fracture identified in the alendronate-treated group (95% CI: 10% to 70%; *p* = 0.021) [29].

The results of the FOSIT trial slightly differed from another famous Fracture Intervention Trial (FIT) performed in the year 1998 for a total duration of four years. The trial included women with low BMD (osteoporotic and osteopenic population) with no prior history of vertebral fractures. In contrast to FOSIT, the FIT study showed the overall reduction in the incidence of fracture in the alendronate group vs. the placebo group (312 vs. 272) but the results were not significant (95% CI: 0.73-1.01) [30]. When seen individually for osteoporotic women (BMD <-2.5) vs. women with comparatively higher BMD, significantly reduced incidence of clinical fractures was noted in the osteoporotic population (95% CI: 0.50-0.82 vs. 95% CI: 0.87-1.35) [30]. The Fracture Intervention Trial Long-term Extension (FLEX) in which subjects of the FIT study treated with alendronate for four years were further randomized into an alendronate group (5 mg/day or 10 mg/day) vs. placebo for another five years. After five years, the cumulative risk of nonvertebral fractures (RR: 1.00; 95% CI: 0.76-1.32) was not significantly different between the two groups (19% and 18.9% in treatment vs. placebo groups). Among those who continued, there was a significantly lower risk of clinically recognized vertebral fractures (5.3% for placebo and 2.4% for alendronate; RR: 0.45; 95% CI: 0.24-0.85) but no significant reduction in morphometric vertebral fractures (11.3% for placebo and 9.8% for alendronate; RR: 0.86; 95% CI: 0.60-1.22) [31]. Post hoc analysis of four controlled randomized trials (BMD Multinational, BMD North America, VERT Multinational, and VERT North America) studied the efficacy of risedronate to reduce fragility fractures in postmenopausal women with osteopenia (i.e., T-scores between -1 and -2.5) with no prevalent vertebral fractures. Treatment with risedronate was found to reduce the risk of morphometric vertebral and non-vertebral fractures by 73% as compared to placebo (95% CI: 0.09-0.83, p: 0.023). The incidence of cumulative non-vertebral fracture was 0.4% in the risedronate group as compared to 5.4% in the placebo-treated group (95% CI: 0.01-0.71, p: 0.022). The cumulative vertebral fracture incidence was 4.25% and 1.8% in the placebo and risedronate group, respectively (95% CI: 0.11-1.78, p: 0.249) [32].

Bisphosphonates were also studied in trials designed for women with a prior history of fractures. A trial conducted by Black et al. included a similar patient population included in the FIT trial, but the difference was the presence of at least one baseline vertebral fracture. At the end of 36 months, the trial concluded that there was a significant reduction of fracture risk in women taking alendronate vs. placebo. This was for both clinical fractures (relative hazard 0.45, 95% Cl: 0.27-0.72) and morphometric vertebral fractures (RR: 0.53, 95% Cl: 0.41-0.68) [33]. The benefits of therapy with bisphosphonate with a prior history of fracture was further tested in a double-blinded study. The subjects were women with a prior history of hip fracture who were treated with yearly zolendronic acid 5 mg after their surgery. The study showed a significant reduction for repeat hip fracture in patients and overall yearly infusion resulted in mortality benefits [34]. The role of bisphosphonate in preventing fracture in women with low BMD and at least one vertebral fracture was further supported by another study performed by Chesnut et al. in the year 2004. The study enrolled more than two thousand five hundred postmenopausal women with a BMD score <-2 at the lumbar spine with at least one vertebra (L1-L4) and one to four prevalent vertebral fractures (T4-L4). The bisphosphonate studied was ibandronate. Patients received either placebo or oral ibandronate administered either daily (2.5 mg) or intermittently (20 mg every other day for 12 doses every three months). The study showed a significant reduction in the incidence of both new morphometric vertebral fracture and clinical vertebral fracture in the ibandronate group (with both daily and intermittent dosing) vs. the placebo group. The risk of non-vertebral fracture, however, remained the same in both groups [35]. Table 1 summarizes the effect of bisphosphonate therapy on fracture risk reduction for patients with osteopenia. 

**Table 1:** Fracture risk reduction with bisphosphonate therapy in osteopenia BMD: bone mass density, CI: confidence interval, FLEX: fracture intervention trial long-term extension, FIT: fracture international trial, FOSIT: Fosamax international trial; HR: hazard ratio, OD: odds ratio, P: Pearson value, RH: relative hazard, RR: relative risk

Study	Drug of choice vs placebo	Duration of study	Subjects	Results (Drug of choice vs placebo)
Reid IR et al. [28]	5 mg intravenous zoledronate once a year.	18 months	Women of age 65 years or older with osteopenia (T score –1 to –2.5).	HR with zolendronic acid for fragility fractures was 0.63; P<0.001. HR with zolendronic acid for non-vertebral fracture was 0.66; P = 0.001. OR for non-vertebral fracture was 0.45; P = 0.002
Pols HA et al. [29] (FOSIT)	10 mg oral alendronate per day.	1 year	Postmenopausal women with BMD <2.	Non-vertebral fracture risk reduction was 47% in the treatment group; P = 0.021
Cummings SR et al. [30] (FIT)	5 mg oral alendronate per day for two years followed by 10 mg per day for the remaining period of the trial.	4.2 years	Women aged 54 to 81 years with a femoral neck BMD of 0.68 g/cm2.	No significant reduction in clinical fractures was noted in the alendronate group for the osteopenic population with RH, 1.08; 95% CI, 0.87-1.35. RR for vertebral fracture with alendronate therapy was 0.56; 95% CI, 0.39-0.80
Black DM et al. [31] (FLEX)	5 mg or 10 mg oral alendronate per day.	5 years	Postmenopausal women who had been randomized to alendronate in FIT, with a mean of 5 years of prior alendronate treatment.	RR for clinically recognized vertebral fractures in the treatment group was 0.45; 95% CI, 0.24-0.85. RR for non-vertebral fractures in the treatment group was 1.00; 95% CI, 0.76-1.32
Posthoc analysis by Siris ES et al. [32]	5 mg oral risedronate per day.	3 years	Postmenopausal women with osteopenia (T score -1.5 to -2.5) were included.	Incidence of cumulative non-vertebral fracture was 0.4%; 95% CI, 0.01-0.71; P = 0.022. Incidence of cumulative vertebral fracture was 1.8% in treatment group; 95% CI, 0.11-1.78; P = 0.249
Black DM et al. [31]	5mg oral alendronate per day for 2 years and then 10mg per day for another one year.	3 years	Women aged 55-81 years with low femoral-neck BMD and at least one existence vertebral fracture.	For vertebral fractures (morphometric) RR was 0.53; 95% Cl, 0.41-0.68. For vertebral fractures (clinical) RH was 0.45 ;95% Cl, 0.27-0.72. For non-vertebral fracture RH was 0.72; 95% Cl, 0.58-0.90
Lyles KW et al. [34]	5 mg intravenous zolendronic acid once a year.	5 years	Men and women 50 years of age or older who had surgical repair of a hip fracture sustained with minimal trauma within 90 days prior to participation in the trial.	The rates of developing new clinical fracture were 8.6% in the treatment group; P = 0.001. The rates of developing a new clinical vertebral fracture were 1.7% in the treatment group; P = 0.02. The rates of developing new non-vertebral fractures were 7.6% in the treatment group; P = 0.03
Chesnut CH et al. [35]	Oral ibandronate administered either daily (2.5 mg per day) or intermittently (20 mg every other day for 12 doses every 3 months).	3 years	Postmenopausal women with a BMD T score	Daily and intermittent dosing of oral ibandronate reduced the risk of new morphometric vertebral fractures by 62% (P = 0.0001) and 50% (P = 0.0006), respectively. Daily and intermittent dosing of oral ibandronate produced a significant RRR in clinical vertebral fractures (49% and 48% for daily and intermittent ibandronate dosing, respectively). The incidence of nonvertebral fractures was similar between the ibandronate and placebo groups after three years (9.1%, 8.9%, and 8.2% in the daily, intermittent, and placebo groups, respectively; the difference between arms not significant).

Effect of bisphosphonates on BMD in osteopenia

Research studies are evident for the positive effect on BMD with bisphosphonate therapy. A double-blind study was conducted on post-menopausal women with a baseline lumbar spine BMD two standard deviations or more below that of the premenopausal women. Oral treatment with alendronate 10 mg for 12 months when compared to the placebo group showed a significant increase in BMD to the lumbar spine, femoral neck, trochanter, and total hip [29]. Besides showing a fracture risk reduction, the FIT trial on women with low BMD also concluded a significant increase in BMD at all sites in patients with no prior history of vertebral fractures taking alendronate 5 mg/day for an average of four years compared to subjects treated with placebo [30]. These results indicate the effect of bisphosphonate treatment on BMD is dose and duration independent. The independence of dose and duration was further explained in a trial conducted by Chestnut et al. Subjects selected were women with a BMD T score -2 or less at the lumbar vertebrae and with at least one prevalent fracture. The bisphosphonate studied was ibandronate administered either daily (2.5 mg) or intermittently (20 mg every other day for 12 doses every three months). The study duration was three years. There were significant and progressive increases in the lumbar spine (6.5%, 5.7%, and 1.3% for daily ibandronate, intermittent ibandronate, and placebo, respectively, at three years) and hip BMD [35]. This study by Chestnut et al. and a study conducted by Delmas et al. [36] showed a decrease in bone turn over with bisphosphonates and a significant reduction in bone turn over markers. Another phase three double-blinded trial performed for a total duration of three years includes treatment of subjects with placebo, alendronate 5 or 10 mg/day for three years or 20 mg/day for two years followed by 5 mg/day for one year. The trial resulted in decreased bone resorption and increased BMD towards the end of the study period [37]. Another study was conducted on postmenopausal women with a normal BMD for placebo vs. daily risedronate 5 mg/day vs. cyclic risedronate for two years duration followed by one year off treatment period. An increase in BMD of lumbar spine and trochanter was observed with both groups of risedronate as compared to the placebo group. In the treatment-free period, there was an increase in bone turnover markers that were associated with a reduction in BMD of the lumbar spine. This concludes that risedronate would maintain BMD when used for a longer duration [38]. Table 2 summarizes the effect of bisphosphonate therapy on BMD in patients with osteopenia.

**Table 2:** Effect of bisphosphonate therapy on bone mass density in osteopenia BMD: bone mass density, FIT: fracture international trial, FOSIT: Fosamax international trial, P = Pearson value

Study	Drug of choice vs placebo	Duration of study	Subjects	Results (Drug of choice vs placebo)
Pols HA et al. [29] (FOSIT)	10mg oral alendronate per day.	1 year	Postmenopausal women with BMD <2.	BMD were significantly (P < 0.001) greater in the treatment group by 4.9% at the lumbar spine, 2.4% at the femoral neck, 3.6% at the trochanter and 3.0% for the total hip.
Cummings SR et al. [30] (FIT)	5 mg oral alendronate per day for two years followed by 10 mg per day for the remaining period of the trial.	4.2 years	Women aged 54 to 81 years with a femoral neck BMD of 0.68 g/cm2.	BMD was significantly (P < 0.001) greater in the treatment group by 3.8% at the femoral neck, 3.4% at the total hip, 8.3% at the lumbar spine.
Chesnut CH et al. [35]	Oral ibandronate administered either daily (2.5 mg per day) or intermittently (20 mg every other day for 12 doses every three months).	3 years	Postmenopausal women with a BMD T score < –2.0 with 1 to 4 prevalent vertebral fractures.	BMD was significantly greater in lumbar spine by 6.5% for daily ibandronate treatment group and 5.7% in an intermittent ibandronate treatment group. A significant increase in hip BMD was also observed.
Mortensen L et al. [38]	Oral risedronate 5 mg per day daily or oral risedronate 5 mg cyclically for two years followed by one year off treatment.	3 years	Early postmenopausal women with normal BMD.	At the end of the second year of the study, the mean increase in BMD of the lumbar spine was 1.4% from baseline in the daily risedronate treatment group and a decrease in lumbar spine BMD of 1.6% in cyclic risedronate treatment group. At the end of the second year of the study, trochanteric BMD at the hip increased by 5.4% in the risedronate 5 mg daily group and by 3.3% in the risedronate 5 mg cyclic group. During one-year treatment-free period bone turnover was increased and lumbar spine BMD was decreased in all three groups.

Cost-effectiveness of treating low BMD with osteopenia

The cost-effectiveness of treating patients with osteoporosis fracture had been established in prior studies. Treating osteoporotic fracture was found to be more cost-effective than treating fragility fractures occurring as a result of non-treatment. The same pattern of cost-effectiveness was observed for treating patients having a prior history of at least one fragility fracture with bisphosphonate [39-41]. On the other hand, when treating postmenopausal women of age 55 to 75 years, having a femoral T-score between -1.5 and -2.4, and with no prior history of fractures pharmacologically, the cost per quality-adjusted life years gained in a study by Schousboe et al. ranged from $70, 000 to $332, 000 with alendronate use. This was found to be clearly not cost-effective when the cost burden of treating with bisphosphonate was compared with the cost of managing acute fractures [42]. Different results were obtained when the cost-effectiveness of drug therapy to prevent osteoporotic fractures in postmenopausal women with osteopenia was analyzed in a Korean population in the year 2016. Drug therapy for osteopenia was found to be a cost-effective intervention depending on the WHO's willingness-to-pay threshold, which is less than the per-capita gross domestic product in Korea (about $25,700) [43]. The population used in that study was similar to the population used in the Schousboe et al. study, which included postmenopausal women with no prior history of fracture. The difference in recommendations between the two studies can be explained by an extensive systemic review conducted by Müller D et al. This study compares the cost-effectiveness of treating osteopenia based upon the fixed threshold of BMD and treating osteopenia based upon BMD and other clinical risk factors. The study concluded that treating a patient would be more cost-effective if the latter approach is utilized. Thus when deciding about initiating treatment for the osteopenic population, clinicians can decrease the cost burden to the healthcare system and provide maximum benefit to the patient [44].

Duration of therapy

There is limited data available regarding the duration of therapy with bisphosphonates and the optimal duration of use has not been determined yet. The FLEX study clearly showed a moderate decline in BMD in those women who discontinued alendronate after five years of treatment but BMD still remains above their pretreatment levels. Risk of fracture did not differ significantly between non-vertebral fractures, but 10-year therapy significantly reduces the risk of having vertebral fractures [31]. Another renowned study, HORIZON-Pivotal Fracture Trial (PFT), compares three versus six years of intravenous bisphosphonate therapy with zoledronic acid. The BMD at all body sites remained constant in women who were continued with zoledronic acid for an additional three years after the initial three years of treatment as compared to those who were in the placebo group after initial three years of treatment and experienced decrease in BMD. The risk for vertebral fracture was found to be significantly lower [45]. The Task Force Report from the year 2016 thus recommends minimal treatment with five years for oral bisphosphonate and three years of intravenous bisphosphonates. For women who are at highest risk of fracture in the near future and have an unstable BMD, it is recommended to continue alendronate or risedronate for 10 years and zoledronic acid for up to six years. For those not at higher risk, a drug holiday of two to three years can be considered, as long term bisphosphonate therapy often increases the risk of atypical femoral fracture but such rare events are outweighed by vertebral fracture risk reduction in high-risk patients [46].

## Conclusions

Many factors need to be considered when placing an osteopenic patient on treatment (T-score: -2.5 to -1). There has been clear data indicating the benefits of placing an osteoporotic patient (T-score: <-2.5) on first-line treatments including bisphosphonates. However, different guidelines give different ideas on which osteopenic patients should be placed on therapy. The pros and cons of treatment must be evaluated. These factors include potential benefits, potential harms, cost of treatment, duration of treatment, life expectancy, and patient preference amongst others. It has been shown that long-term usage of first-line treatments can potentially reduce the risk of future fractures. However, it is still unclear whether or not osteopenic patients should definitively.
